# Patient willingness to pay and preference for cervical cancer treatments among middle- and low-income populations in Xinjiang

**DOI:** 10.1186/s41687-025-00938-6

**Published:** 2025-08-21

**Authors:** Lina Zhu, Yan Wang, Shangjie Yang, Qianhui Li, Jie Wang, Jun Zhao, Jianhua Wang, Yubo Wang

**Affiliations:** 1 Department of Pharmacy, Xinjiang Production and Construction Corps Hospital, Urumqi, 830000 China; 2 Office of Cancer Prevention and Treatment Research, Affiliated Tumour Hospital of Xinjiang Medical University, Urumqi, 830000 China; 3 School of Pharmacy, Xinjiang Medical University, Urumqi, 830054 China; 4 Department of Pharmacy, First Affiliated Hospital of Xinjiang Medical University, Urumqi, 830011 China; 5 Xinjiang Key Laboratory of Clinical Drug Research, First Affiliated Hospital of Xinjiang Medical University, Urumqi, 830011 China

**Keywords:** Cervical cancer, Health utility value, Quality of life, Willingness to pay

## Abstract

**Background:**

Cervical cancer remains a significant public health issue in underdeveloped regions like Xinjiang, Western China, where health literacy is low and economic disparities are prominent. While previous studies have focused on preventive measures, there is limited research on the willingness to pay (WTP) for cervical cancer treatments. This study aimed to assess patient preferences and WTP from patient perspective for quality of life improvement, unadjusted life-year extension, and targeted and immunotherapy drugs among cervical cancer patients in Xinjiang.

**Methods:**

A face-to-face survey was conducted using the Contingent Valuation Method (CVM) and Discrete Choice Experiment (DCE) to evaluate WTP for cervical cancer treatments. The CVM assessed patients’ WTP for two scenarios: living in perfect health for 5 or 10 years versus unadjusted life-year extension for the same durations. Health related quality of life (HRQoL) was measured using both the EQ-5D-5 L and EQ-VAS instruments. The DCE evaluated patients’ preferences and WTP for targeted therapy and immunotherapy drugs.

**Results:**

This study included 106 valid questionnaires (response rate 96.4%), primarily comprising stage III cervical cancer patients (EQ-5D-5 L 0.89, EQ-VAS 0.80) with characteristics of low income and educational attainment. Key findings revealed: (1) Patients prioritized quality of life improvement over lifespan extension, with WTP/QALY in the 10-year perfect health scenario exceeding Xinjiang’s 2024 per capita disposable income threshold while other scenarios remained below this value, and WTP/QALY being significantly higher in the 10-year scenario compared to the 5-year scenario; (2) Medication preference analysis demonstrated that quality of life improvement, cost, and incidence of adverse reactions were key decision-making factors, whereas survival extension held relatively lower importance. WTP valuations exhibited significant attribute-specific variations, with the highest WTP corresponding to quality of life improvement attributes and the lowest to survival extension attributes.

**Conclusions:**

Cervical cancer patients in Xinjiang prioritize quality of life improvement over lifespan extension, with their preferences and WTP being influenced by treatment-related factors, disease characteristics, and socioeconomic background. Therefore, when formulating reimbursement policies and resource allocation strategies, priority should be given to interventions that can significantly improve quality of life, while implementing differentiated support policies for patient populations with varying socioeconomic statuses.

**Supplementary Information:**

The online version contains supplementary material available at 10.1186/s41687-025-00938-6.

## Background

Cervical cancer (CCA) ranks second in age-adjusted incidence and mortality rates among female-specific cancers, second only to breast cancer [1], accounting for 18% and 17% of global incidence and mortality rates, respectively [2] according to data from the National Cancer Center of China in 2022. Treatment options for cervical cancer patients include traditional surgery, radiotherapy, chemotherapy, and newly developed therapies with significant efficacy and effectiveness, such as targeted therapy and immunotherapy. The choice and effectiveness of these treatments are directly related to patients’ health outcomes and economic burdens [3].

Although China’s medical insurance covers 43%-55% of direct medical costs for cervical cancer patients, they still face high out-of-pocket expenses [4]. A 2018 survey in Henan Province [5] found that total costs per patient—from diagnosis to one-year post-discharge—ranged from USD 8,066 to 22,888 (based on USD/CNY exchange rates at the time), with quality adjusted life year (QALY) losses between 0.05 and 0.26. Amid rising medical costs, rational resource allocation has grown increasingly critical under constrained healthcare budgets. Cost-effectiveness thresholds (CETs), a key evaluation metric for healthcare interventions, are currently defined in China as 1–3 times per capita GDP from the healthcare perspective. While CETs primarily function as supply-side budget constraints, their theoretical basis and applicability remain academically debated [6]. In recent years, there has been a growing recognition of willingness to pay per QALY (WTP/QALY) as an efficient approach to optimizing marginal efficiency across healthcare systems [7]. In China, WTP/QALY thresholds have been studied for cancers like lung and breast cancer [8, 9], offering optimized evidence-based guidance for resource allocation. Exploring cervical cancer patients’ WTP can provide crucial insights for resource allocation [10].

WTP is a key economic indicator for evaluating QALY and assessing demand-side preferences. WTP reflects the societal valuation of health by quantifying individuals’ WTP for health improvements, such as enhanced quality of life or extended lifespan, rooted in welfare economics principles [11]. The Contingent Valuation Method (CVM) and the Discrete Choice Experiment (DCE) were employed to elicit patients’ WTP and preferences. CVM estimates WTP in hypothetical market scenarios based on the utility theory of welfare economics, whereas DCE gathers preferences through attribute-based choice tasks grounded in random-utility theory and random-utility maximization [12, 13]. These methods have been used to explore WTP to the quality of life improvements or life-year extensions for targeted and immunotherapy drugs among patients with various cancers, including lung cancer [14–17], breast cancer [9, 18, 19], and colorectal cancers [20, 21]. However, research on WTP among cervical cancer patients, especially regarding treatment, remains limited [22].

A systematic review of global literature on WTP for cervical cancer treatment identified one study by Lang HC et al. (2010) [22]. The Lang HC et al.’s study (2010) [22] employed the CVM method to assess the WTP for complete remission of cervical cancer in Chinese patients, and rest of the studies focused almost exclusively on preventive strategies such as HPV vaccination and screening. Since then, research has predominantly focused on preventive measures for cervical cancer, with limited attention to WTP for treatment. The Xinjiang Uygur Autonomous Region in northwestern China, a vast and remote area with diverse ethnic groups, including Uyghurs, Han, and Kazakhs, and others. It has a higher cervical cancer detection rate than the national average and lower health literacy [23, 24]. Given the region’s economic conditions, low health literacy, and high incidence of cervical cancer, studying WTP among patients here is critical.

Employing a joint CVM–DCE design, this study quantifies cervical cancer patients’ WTP for perfect health and extended survival, and ranks the attributes that drive their preferences for novel targeted or immunotherapy drugs. Specifically, it addresses (1) the magnitude of WTP under hypothetical treatment scenarios within this population, and (2) the key attributes determining drug choice and their relative importance. The resulting estimates begin to fill the Northwest China evidence gap and offer policymakers and manufacturers concrete data on affordability and patient-valued benefits when setting prices or coverage.

## Methods

**Fig. 1:**
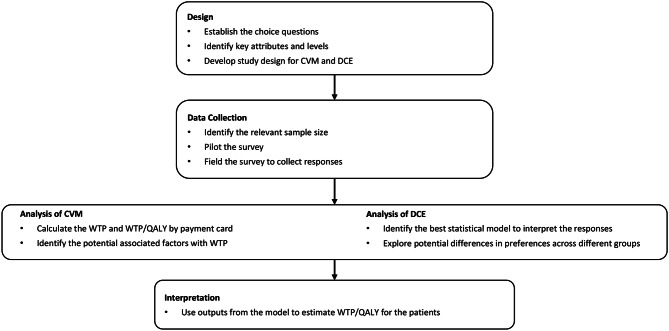
Methodological steps to perform and analyze contingent valuation method and discrete choice experiment

Figure 1 presents the design, application, and analysis of CVM and DCE involve methodological steps, summarized below and informed by previously published guidelines [12, 25].

### Data source

This study recruited cervical cancer patients from two major tertiary hospitals in Xinjiang—a general teaching hospital and a specialized cancer center—serving patients from across the region. Although only two hospitals were selected, they represent the top-tier general teaching hospital and specialized cancer center in Northwest China, serving patients from all regions of Xinjiang. We therefore believe the sample demonstrates good generalizability, from June to August 2024. Inclusion criteria were as follows: (1) confirmed diagnosis of cervical malignancy; (2) age ≥ 18 years; (3) patient with a willingness to participate in the study by verbal informed consent. Exclusion criteria were: (1) patients with precancerous cervical lesions; (2) patients with cognitive impairments that hinder understanding of the questionnaire.

### Study design

#### Contingent valuation method

This study developed two health improvement scenarios to assess patient preferences for hypothetical drugs: Scenario one focused on quality of life improvement, and Scenario two focused on unadjusted life-year extension. Scenario one evaluated patients’ WTP to maintain a perfect health state (health utility value = 1) for 5 or 10 years. Scenario two assessed WTP for extending lifespan with drugs effective for 5 or 10 years in the current health state. WTP was measured using the payment card method [26], which provided a range of preset payment amounts based on China’s 2023 per capita GDP [27], from 0.1 to 10 times the GDP. Patients selected the range and maximum amount they were willing to pay. The CVM design flowchart is shown in Appendix 1. The study measured HRQoL using both the EQ-5D-5 L [28] (with the Chinese tariff) and the EQ-VAS. While the EQ-5D-5 L generates population preference-based utility values, the EQ-VAS captures direct patient-reported health assessments. This dual approach enables a comparison of societal (population-level) and individual (patient-level) perspectives when evaluating WTP for cervical cancer treatment [28, 29]. WTP/QALY was determined by combining patients’ WTP with the corresponding QALY values.

#### Discrete choice experiment

Attributes and levels for targeted drugs and immunotherapy in cervical cancer were identified through literature reviews [30, 31], focus group discussions and three-round expert consultations. Consultations with 13 oncology experts and 15 cervical cancer patients provided feedback on attribute importance and understanding (detailed determination of attributes and levels for DCE in Supplementary File Appendix 2). Five attributes, each with three levels, were identified, leading to 243 (3⁵) theoretical options. An orthogonal design was used to create the questionnaire, balancing respondent burden and data requirements. This approach generated ten choice sets, each containing three options. Attribute selection was based on literature review and qualitative research, with five core attributes ultimately identified through focus group discussions involving 13 experts and 15 patients. Although quantitative ranking of attribute importance was not employed, iterative discussions ensured all selected attributes were jointly endorsed by both participant groups. The relative D-efficiency of the design was 97.33%, indicating a high-quality design. This design ensured scientific rigor while managing respondent cognitive load. Specific attributes and levels were further refined through reviews of clinical trials on cervical cancer targeted and immunotherapy drugs [32–36]. Drug prices were determined by examining market prices, and the medical reimbursement rate was set according to Chinese healthcare policies. The study covered clinical ranges and potential extremes for each attribute. Table 1 presents the Scenarios of CVM and an Example Choice Task of DCE.

**Table 1:**
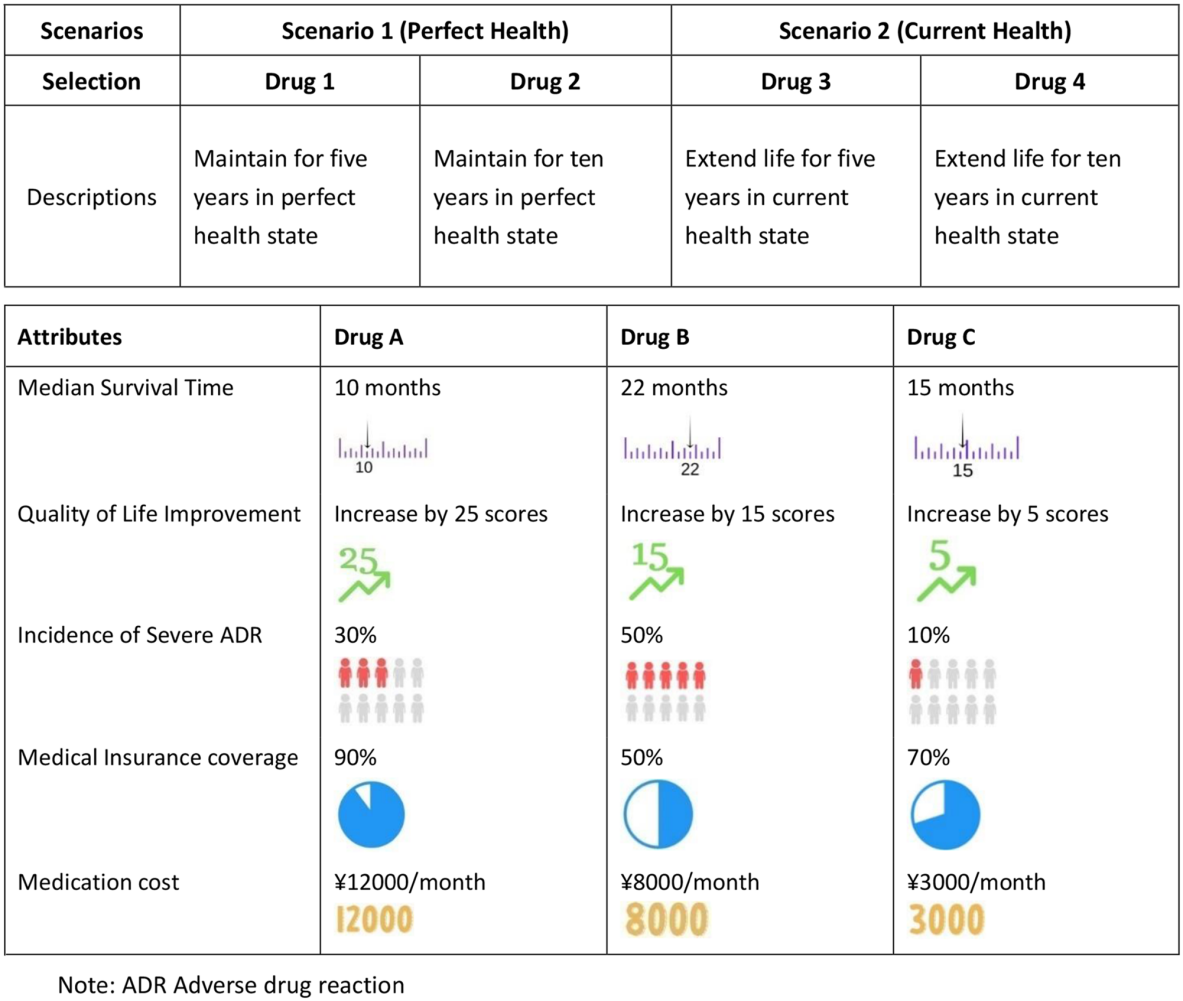
Scenarios of contingent valuation methods and an example choice task of discrete choice experiment

#### Questionnaire design

The research questionnaire was divided into three main sections. The first section collects patient personal information, including the hospital visited, patient ID, average annual income, and level of family support. Based on the CVM, the second section includes questions related to WTP, such as the EQ-5D-5 L, EQ-VAS, WTP for quality of life improvement, and WTP for life extension. The third section uses DCE methodology to address drug preferences, including familiarity with targeted and immunotherapy drugs, understanding of the questions, attribute importance ranking, and 11 choice sets (10 primary and one quality control). A pretest was conducted to assess the questionnaire’s clarity, and adjustments were made based on feedback to improve its effectiveness. (The complete questionnaire is available in Supplementary File Appendix 3).

### Data collection and quality control

The minimum sample size for this study was 50 participants, calculated using the method proposed by Johnson and Orme [37]. Data was collected through face-to-face interviews with trained interviewers from relevant educational backgrounds. To ensure data quality, the CVM design included a negative option (“I am not willing to pay for this”) to minimize bias in WTP estimates. In the DCE design, quality control questions were included, and patients who answered incorrectly were excluded, indicating their inability to understand the questionnaire.

$$\:N>\frac{500c}{t\times\:a}$$

In Johnson and Orme’s rule, *N* denotes the required sample size, 500 is treated as a fixed constant, *c* equals the maximum number of levels for any single attribute, *t* is the number of choice sets presented to each respondent, and *a* is the number of alternatives per choice set. For the present study, *c* = 3, *t* = 10, and *a* = 3. With these parameters, a sample of more than 50 cervical cancer patients satisfies the minimum requirement.

Patients’ WTP and QALY data were collected via questionnaires. QALY values were calculated using the EQ-5D-5 L Chinese population utility system [28] and EQ-VAS to reflect general and individual health levels. Personal information, such as age, occupation, and educational level, was obtained from hospital electronic medical records, and all data were anonymized during processing to protect patient privacy.

### Statistical analysis

Double-independent verification (ZLN & YW) and data cleaning ensured data accuracy and consistency. Descriptive statistical analysis was used to report categorical variables as frequencies and percentages. For continuous variables, means ± standard deviations (SD) were reported for normally distributed data, while medians and interquartile ranges (IQR) were used for non-normally distributed data. The WTP was reported in the Chinese Yuan (CNY) and exchanged to US dollars (USD) in the currency exchange rate in August 2024 (1 CNY = 0.14 USD). The survey questionnaire database was established using Microsoft Excel^®^ 2019, and all statistical analyses were performed with Stata 14.0.

The CVM study reported the maximum WTP (WTP_max_), average WTP (WTP_average_), and their frequency distributions for quality of life improvement and life extension, as well as QALY related WTP/QALY values. WTP_max_ was defined as the highest value within the payment interval. Backward stepwise linear regression (with a removal threshold of *P* ≥ 0.05) was used to analyze factors associated with WTP/QALY. WTP/QALY for quality of life (WTP/QALY quality) represented the WTP for one year of life in perfect health. In comparison, WTP/QALY for life extension (WTP/QALY length) represented the WTP for one additional year of life in the current health state. WTP/QALY was calculated using the following formula:

$$\eqalign{& WTP/QALY \cr& quality = {{WTP} \over {(1 - Current\>Heatlh\>Utility) \times Time}} \cr} $$

$$\eqalign{& WTP/QALY \cr& length = {{WTP} \over {Current\>Heatlh\>Utility \times \>Time}} \cr} $$

Which *Time* was fixed at either 5 or 10 years, *WTP* estimate reflects an immediate, lump-sum payment with no discounting applied.

The DCE study used a Logit model to analyze patient preferences for different attributes and levels. The most suitable model was chosen by comparing the Akaike Information Criterion (AIC) and Bayesian Information Criterion (BIC) values of the conditional and mixed Logit models, with smaller values indicating a better model. Drug and patient out-of-pocket costs were included as independent variables, calculated as drug price × (1 - reimbursement rate). This approach reflects patient WTP and decision-making tendencies under various economic conditions, providing more accurate estimates of marginal WTP (MWTP). A conditional logit model was conducted initially to assess the preference levels of various attributes in the DCE, which adopted dummy variables to capture the impact of different attribute levels on choices. Subsequently, the Randomization Inference (RI) was applied to evaluate the importance of each attribute based on changes in the log-likelihood ratio. The model used effect coding variables to ensure an accurate measurement of the relative impact of each attribute level. MWTP and RI were calculated using the following formulas:

$$\:MWTP=-\frac{\beta\:xk}{{\beta\:}_{cost}}$$

$$\:RI=\frac{\varDelta\:\beta\:xt}{\sum\:\varDelta\:\beta\:}\times\:100\%$$

Where β is the regression coefficient, x is the attribute, and k is the level. The difference between each attribute’s maximum and minimum regression coefficients is Δβxk.

### Ethical approval

The study adhered to good clinical practice guidelines and the Declaration of Helsinki. This study was approved by the First Affiliated Hospital of Xinjiang Medical University Research Ethics Committee (REF: K202401-01). All research materials (including informed consent forms and questionnaires) in this study were provided in standardized bilingual versions (Chinese and Uyghur), with trained bilingual researchers providing one-on-one guidance to participants throughout the process.

## Results

Pilot testing was conducted with fifteen cervical cancer patients, twelve of whom demonstrated full comprehension and provided valid responses. Based on feedback from four participants, we implemented the following improvements: (1) adjusted the willingness-to-pay (WTP) bid ranges to GDP-based intervals; (2) restated DCE attributes and levels using more lay clinical terminology. In the formal survey, 110 questionnaires were collected, and 106 valid responses were included in the analysis, with a response rate of 96.4%.

### Characteristics of the respondents

The sample was predominantly Han (55.7%) and had low education levels, with 55.7% having education at or below primary school. Most patients had annual incomes below the Xinjiang average [38], and the average age was 56. Most were at stage III of cervical cancer with a disease duration of less than three months. The median EQ-5D-5 L health utility value was 0.89, and the median EQ-VAS score was 0.80. Only four patients received targeted therapy or immunotherapy, with bevacizumab and camrelizumab being the primary drugs. Detailed characteristics of the patients are shown in Table 2.

**Table 2:** Characteristics of cervical cancer patients responding to the questionnaire (*n* = 106)

Variables	Number	Percentage	Variables	Number	Percentage
Age (mean ± SD)	55.9 ± 1.2		Annual income level		
EQ-5D-5 L (median, IQR)	0.89(0.78, 0.95)		Below¥12,000	12	11.3%
EQ-VAS (median, IQR)	0.80(0.60, 0.90)		¥12,000 -¥30,000	13	12.3%
Number of children (median, IQR)	2(1, 4)		¥30,000 -¥50,000	8	7.6%
Age-adjusted CCI (median, IQR)	5(3, 7)		¥50,000 -¥70,000	10	9.4%
**Ethnicity**			¥70,000 -¥100,000	6	5.7%
Han	59	55.7%	Above¥100,000	2	1.9%
Minorities	47	44.3%	No work, no income due to illness	9	8.5%
**Marital status**			No work, no income	46	43.4%
Married	88	83.0%	**Duration of illness**		
Other	18	17.0%	Less than three months	75	70.8%
**Occupation**			More than three months	31	29.2%
Retired	14	13.2%	**Whether first-time treatment**		
Unemployed	25	23.6%	Yes	49	46.2%
Employee	10	9.4%	No	57	53.8%
Farmer	18	17.0%	**Duration of treatment**		
Self-employed	9	8.5%	Less than three months	80	75.5%
Other	30	28.3%	More than three months	26	24.5%
**Residence**			**Whether received immediate treatment**		
Northern Xinjiang	62	58.5%	Yes	99	93.4%
Southern Xinjiang	44	41.5%	No	7	6.6%
**Educational level**			**Previous treatment methods**		
Primary school or below	59	55.7%	First visit to the hospital for treatment	49	46.2%
Junior high school	25	23.6%	Single treatment	24	22.6%
High school (including vocational school)	11	10.4%	Combination treatment	33	31.1%
University (college and above)	11	10.4%	**Current treatment methods**		
**Cervical cancer stage**			Single treatment (medication/radiotherapy)	44	41.5%
Stage I	12	11.3%	Combination treatment (medication + radiotherapy)	62	58.5%
Stage II	23	21.7%	**Whether recurrence**		
Stage III	60	56.6%	Yes	2	1.9%
Stage IV	11	10.4%	No	104	98.1%

Note: SD: Standard deviation CCI: Charlson Comorbidity Index; Ethnic minorities, including Uygur, Kazakh, Hui, and Tajik ethnics; Immediate treatment is defined as treatment duration of less than three months

### Results of the contingent valuation method

#### Willingness to pay

**Fig. 2:**
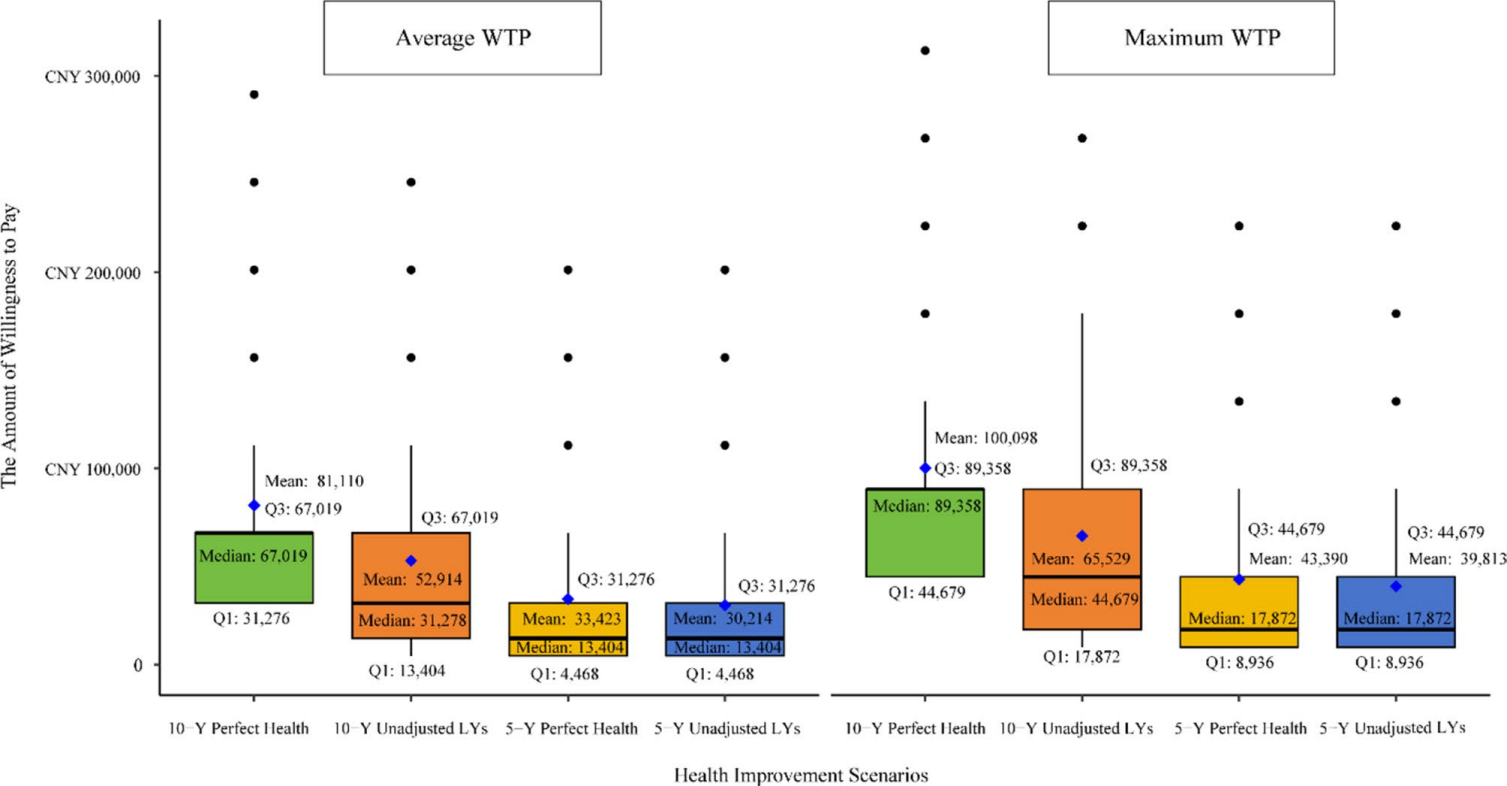
WTP_max_ and WTP_average_ in Perfect Health Scenario (*n* = 104) and Life Extension Scenario (*n* = 102). Note: WTP_max_ reflects the highest willingness to pay selected by patients in the questionnaire, representing the maximum amount patients are willing to pay under ideal conditions. WTP_average_ indicates the average willingness to pay, providing a more representative and stable measure. The black dots in the figure represent outliers or extreme values. “5-Y Perfect Health” refers to a 5-year living in perfect health; “10-Y Perfect Health” refers to a 10-year living in perfect health; “5-Y Unadjusted LYs " refers to a 5-year survival gain without change in health utility; and “10-Y Unadjusted LYs " refers to a 10-year survival gain without change in health utility

Of the 106 responses, two patients were unwilling to pay for perfect health restoration, and four were unwilling to pay for life extension. Detailed WTP amounts for various scenarios are reported in Fig. 2.

In the “10 years of perfect health” scenario, patients showed the highest WTP. The median WTP_max_ was CNY 89,358 (IQR 44,679 − 89,358), approximately USD 12,563.73 (IQR 6,281.87-12,563.73). The median WTP_average_ was CNY 67,019 (IQR 31,276 − 81,110), approximately USD 9,435.53 (IQR 4,397.41-11,404.07), reflecting a high valuation of long-term perfect health. The mean WTP_max_ reached CNY 100,636 (USD 14,149.42), indicating a high WTP among some patients. Conversely, in the “10-years of unadjusted life-year extension” scenario, WTP was significantly lower, with a median WTP_max_ of CNY 44,679 (IQR 17,872 − 89,358), approximately USD 6,281.87 (IQR 2,512.80–12,563.73), and a median WTP_average_ of CNY 31,276 (IQR 13,404 − 67,019), approximately USD 4,397.41 (IQR 1,884.60-9,422.87), while the mean WTPmax was CNY 65,529 (USD 9,213.38).

For the “5 years of perfect health” and “5 years of unadjusted life-year extension” scenarios, WTP was relatively low and similar, with WTP_max_ at CNY 8,936 (USD 1,256.40) and WTP_average_ at CNY 4,468 (USD 628.20) for both. The WTP_max_ was slightly higher for the 5-year perfect health scenario than the unadjusted life-year extension scenario.

#### WTP of health improvement per QALY

Table 3 presents the WTP_max_/QALY and WTP_average_/QALY amounts patients are willing to pay for different health improvement scenarios. Patients consistently attach a higher WTP/QALY to quality of life improvement than to unadjusted life-year extension, but WTP/QALY varies with both the utility instrument and the time horizon. Under EQ-5D-5 L, the 10-year perfect-health scenario elicits roughly double the WTP/QALY of its five-year counterpart, whereas the valuations for unadjusted life-year extension showed no statistically significant difference between the two-time horizons. In contrast, the EQ-VAS yields a 2.5-fold increase from five to ten years in the perfect health scenario, and assigns slightly higher, yet time-stable, values to life-year extension, underscoring the instrument’s greater weight on patients’ subjective valuation of survival gains.

**Table 3:** WTP/QALY of cervical cancer patients in different health improvement scenarios (Median, IQR)

Health improvement scenarios	Quality of life improvement		Unadjusted life-year extension	
	5-Year perfect health state	10-year perfect health state	5-year unadjusted life-years	10-year unadjusted life-years
EQ-5D-5 L Utility	*n* = 83		*n* = 99	*n* = 100
WTP max/QALY	CNY 33,338(13,989, 73,456)	CNY 64,432(29,407, 115,337)	CNY 4,149(2,678, 10,177)	CNY 4,700(2,030, 10,336)
WTP average/QALY	CNY 25,004(9,948, 54,274)	CNY 48,868(21,600, 102,189)	CNY 3,111(1,340, 7,259)	CNY 3,290(1,523, 7,598)
EQ-VAS Utility	*n* = 91		*n* = 101	*n* = 102
WTP max/QALY	CNY 17,872(7,149, 44,679)	CNY 44,679(17,872, 61,670)	CNY 5,957(3,574 11,170)	CNY 4,964(2,314 11,170)
WTP average/QALY	CNY 13,404(5,362, 41,421)	CNY 31,276(13,404, 53,056)	CNY 4,468(1,787, 7,819)	CNY 3,475(1,735, 8,377)

Table 4 presents pairwise significance tests of differences in WTP/QALY across health state scenarios and utility instruments. Significant differences in WTP/QALY were observed between the 10-year and 5-year perfect health scenarios (*P* < 0.001). However, in the unadjusted life-year extension scenario, differences between the 10-year and 5-year WTP/QALY were not significant (EQ-5D: *P* = 0.936; EQ-VAS: *P* = 0.852). This finding corroborates the results presented in Table 3.

**Table 4:** Pairwise significance tests of differences in WTP_average_/QALY across health state scenarios and utility instruments

WTP average/QALY	10Y_PH_EQ5D	5Y_PH_EQ5D	10Y_PH_VAS	5Y_PH_VAS	5Y_LY_VAS	5Y_LY_EQ5D	10Y_LY_VAS	10Y_LY_EQ5D
10Y_PH_EQ5D	-	< 0.001***	< 0.001***	< 0.001***	< 0.001***	< 0.001***	< 0.001***	< 0.001***
5Y_PH_EQ5D		-	0.085	< 0.001***	< 0.001***	< 0.001***	< 0.001***	< 0.001***
10Y_PH_VAS			-	< 0.001***	< 0.001***	< 0.001***	< 0.001***	< 0.001***
5Y_PH_VAS				-	< 0.001***	< 0.001***	< 0.001***	< 0.001***
5Y_LY_VAS					-	< 0.001***	0.852	< 0.001***
5Y_LY_EQ5D						-	0.327	0.936
10Y_LY_VAS							-	< 0.001***
10Y_LY_EQ5D								-

Note: Since the WTP average/QALY values do not satisfy normality assumptions, non-parametric rank-sum tests were employed for analysis. *** denotes *P* < 0.001. Y: years; PH: Perfect Health Scenario; LY: the Unadjusted Life-Years Scenario; EQ-5D and EQ-VAS refer to the EQ-5D-5 L and EQ-VAS measurement tools, respectively

### Results of discrete choice experiment

The conditional logit model was adopted the mixed logit estimation repeatedly returned non-concavity warnings and, given the limited sample size, could not reliably capture preference heterogeneity. The regression results revealed no inherent preference differences among patients for various drug options without considering specific attributes.

Table 5 presents cervical cancer patients’ preferences for each medication attribute level were statistically significant (*P* < 0.05). Shorter median survival times of 10 and 15 months significantly reduced patient preferences compared to the 22-month baseline (10-month: OR 0.68, 95%CI 0.57, 0.81; 15-month: OR 0.71, 95%CI 0.59, 0.84; both *P* < 0.001). Improvements in quality of life scores and reductions in severe adverse events had a more substantial impact on choices. Increasing quality of life scores from 5 to 15 points significantly enhanced preferences (OR 1.38, 95% CI 1.05–1.81, *P* < 0.005), and increasing from 5 to 25 points further enhanced preferences (OR 2.40, 95% CI 1.98–2.90, *P* < 0.005), while reducing adverse event rates from 50 to 30% significantly improved preferences (OR 1.60, 95% CI 1.29–1.99, *P* < 0.001), and further reduction to 10% showed greater improvement (OR 2.28, 95% CI 1.86–2.80, *P* < 0.001). Although economic factors like reimbursement rates and drug prices influenced preferences, clinical attributes had a more significant effect. Patients demonstrated significantly lower preferences for reduced reimbursement rates compared to the 90% reference level (50% rate: OR 0.75, 95% CI 0.61–0.92; 70% rate: OR 0.78, 95% CI 0.66–0.94; both *P* < 0.01).

**Table 5:** Preferences for each medication attribute level among cervical cancer patients based on conditional logit model (*n* = 106)

Attributes and Levels	Coefficient (SE)	Coefficient 95%CI	Odds Ratio (SE)	Odds Ratio 95%CI	*P*
ASC1	-0.59(0.14)	(-0.33, 0.21)	0.94(0.13)	(0.72, 1.24)	0.671
ASC2	0.04(0.16)	(-0.27, 0.35)	1.04(0.17)	(0.76, 1.43)	0.796
Median Survival Time (Reference: 22 months)					
10 months	-0.39(0.09)	(-0.57, -0.21)	0.68(0.06)	(0.57, 0.81)	< 0.001
15 months	-0.35(0.09)	(-0.53, -0.17)	0.71(0.06)	(0.59, 0.84)	< 0.001
Quality of Life Improvement (Reference: 5 points)					
15 points	0.32(0.14)	(0.05, 0.59)	1.38(0.19)	(1.05, 1.81)	0.002
25 points	0.87 (0.10)	(0.68, 1.07)	2.40(0.24)	(1.98, 2.90)	< 0.001
Incidence of Severe Adverse Reactions (Reference: 50%)					
10%	0.82(0.10)	(0.62, 1.03)	2.28(0.24)	(1.86, 2.79)	< 0.001
30%	0.47(0.11)	(0.25, 0.69)	1.60(0.18)	(1.29, 1.99)	< 0.001
Insurance Reimbursement Rate (Reference: 90%)					
50%	-0.29(0.10)	(-0.49, -0.09)	0.75(0.08)	(0.61, 0.92)	0.005
70%	-0.24(0.09)	(-0.42, -0.07)	0.78(0.07)	(0.66, 0.94)	0.007
Drug Cost (Reference: CNY 12,000)					
CNY 3,000	0.92(0.12)	(0.70, 1.15)	2.51(0.29)	(2.00, 3.15)	< 0.001
CNY 8,000	0.50(0.10)	(0.31, 0.69)	1.65(0.16)	(1.37, 2.00)	< 0.001
Log Likelihood	-1005.90				
AIC, BIC	2034.62, 2040.44				
LR Chi-square	318.43***				
Pseudo R^2^	0.14				
Observations	3180				

Note: SE: Standard Error; 95%CI: 95% Confident Interval; CNY: Chinese Yuan; ASC: Alternative Specific Constants; AIC: Akaike Information Criterion; BIC: Bayesian Information Criterion

ASC1 and ASC2 are alternative specific constants (ASCs) used to capture the systematic preferences for each option that are not accounted for by other explanatory variables. ASC_1 represents the inherent preference for Option B relative to Option A, while ASC_2 represents the inherent preference for Option C relative to Option A. Since both coefficients are not significant (*P* > 0.05), this indicates that, without considering other drug attributes, patients do not exhibit significant preference differences between Options B and C compared to Option A; *** denotes *P* < 0.001

#### Willingness to pay for marginal drug attributes

Table 6 presents the MWTP among cervical cancer patients: the greatest MWTP is observed for improvements in quality of life scores, followed by reductions in the severity of adverse reactions, increases in median survival time, and higher insurance reimbursement rates. Table 7 reports the relative importance of medication attributes derived from partial log-likelihood analysis, with quality of life improvement ranked as the most influential attribute, followed by drug cost.

**Table 6:** Marginal willingness to pay and Out-of-Pocket costs for each medication attribute level among cervical cancer Patients(*n* = 106)

Attributes and Levels	WTP for Medication (SE)	WTP for Medication 95%CI	Out-of-Pocket WTP (SE)	Out-of-Pocket WTP 95%CI
Median Survival Time (Reference: 22 months)				
10 months	- CNY 4,041(854) ***	(-5,717, -2,366)	- CNY 2,163(451) ***	(-3,046, -1,280)
15 months	- CNY 3,568(926) ***	(-5,454, -1,682)	- CNY 2,133(492) ***	(-3,098, -1,167)
Quality of Life Improvement (Reference: 5 points)				
15 points	CNY 2,771(1,445)	(-61, 5,605)	CNY 2,478(649) ***	(1,205, 3,751)
25 points	CNY 8,475(1,383) ***	(5,766, 11,185)	CNY 4,504(579) ***	(3,368, 5,639)
Incidence of Severe Adverse Reactions (Reference: 50%)				
10%	CNY 8,212(1,080) ***	(6,095, 10,330)	CNY 3,560(573) ***	(2,437, 4,682)
30%	CNY 4,729(944) ***	(2,879, 6,579)	CNY 1,476(477) **	(541, 2,410)
Insurance Reimbursement Rate (Reference: 90%)				
50%	- CNY 2,630(1,127) *	(-4,839, -422)	-	-
70%	- CNY 2,213(862) *	(-3,901, -524)	-	-

Note: Willingness to pay is expressed in RMB/month; WTP for Medication represents the total amount patients are willing to pay for changes in drug attributes without considering the reimbursement ratio of health insurance. Out-of-Pocket WTP: represents the amount patients are willing to pay after considering the reimbursement ratio of health insurance. * indicates *P* < 0.05, ** indicates *P* < 0.01, and *** indicates *P* < 0.001. CI: Confidence interval

**Table 7:** Relative importance of medication attributes derived from partial Log-Likelihood analysis

Attribute level excluded from the analysis	Log-likelihood	Partial effect-change in log-likelihood	The relative effect-% sum of the change in log-likelihood	Cumulative (%)	Order of impact
None (full model)	-1005.31				
Quality of Life Improvement	-1057.87	52.56	37.4%	37.4%	1
Drug Cost	-1041.78	36.46	25.9%	63.3%	2
Incidence of Severe Adverse Reactions	-1040.25	34.94	24.9%	88.2%	3
Median Survival Time	-1016.37	11.06	7.9%	96.1%	4
Insurance Reimbursement Rate	-1010.86	5.54	3.9%	100.0%	5

For median survival time, both the 10-month and 15-month periods significantly reduce WTP compared to the 22-month baseline. The marginal out-of-pocket WTP decreases by CNY 2,163/month (USD 304.12) when survival is reduced from 22 to 10 months and by CNY 2,133/month (USD 299.90) for a reduction to 15 months, with only a CNY 30/month (USD 4.22) difference between the two. The total WTP decreases by CNY 4,041/month (USD 568.16) for a reduction from 22 to 10 months, but patients are still willing to pay more for any survival extension.

For quality of life improvements, increasing the score from 5 to 25 points leads to a total WTP of CNY 8,475/month and an out-of-pocket WTP of CNY 4,504/month (USD 633.26) (*P* < 0.001), indicating strong preferences for quality of life improvement. Reducing adverse reaction rates from 50 to 10% results in a total WTP of CNY 8,212/month (USD 1,154.61) and an out-of-pocket WTP of CNY 3,560/month (USD 500.54) (*P* < 0.001). When the rate is reduced to 30%, the WTP of the total is CNY 4,729/month (USD 664.90), with an out-of-pocket WTP of CNY 1,476/month (USD 207.53) (*P* < 0.01).

## Discussion

The cervical cancer patient population in Xinjiang exhibits distinct socioeconomic characteristics, including middle- and low-income levels, limited education, and a high proportion of advanced-stage cases. These demographic patterns highlight three major challenges in cancer prevention and treatment in western China: disease-induced economic vulnerability, delayed medical seeking due to limited health literacy [23, 24], and diagnostic delays exacerbated by insufficient healthcare resources. This unique population profile provides valuable insights for examining health preferences in economically underdeveloped areas, particularly offering special significance for assessing WTP measures.

The study yielded three key findings: First, patients systematically valued quality of life improvement more highly than survival extension, a phenomenon that contrasts with some existing studies. Second, WTP demonstrated a distinct socioeconomic gradient, significantly influenced by clinical stage, income level, and educational attainment. Notably, quality of life improvement, treatment cost, and adverse effect risk emerged as the core dimensions in patients’ treatment decision-making process.

In the context of cervical cancer treatment in China, patients still have to bear out-of-pocket payments ranging from several thousand to tens of thousands of RMB, even though the bulk of costs is covered by the public insurance schemes. Consequently, price signals are not entirely absent. Yet, because reimbursement ratios are high and prices are largely administratively set, market prices fail to fully reflect the true scarcity of resources. Under these circumstances, direct observation of market behavior is liable to under- or over-estimate genuine WTP. Experimental WTP estimates can therefore serve as complementary evidence to help assess the economic rationale for aligning additional social investment with patients’ personal financial burden [39]. Cervical cancer patients’ WTP/QALY values were significantly lower than those in other studies. Values ranged from 0.04 to 1.04 times the per capita GDP, compared to 1.94 times GDP for end-stage disease patients in China [40], 1.16 times GDP for malignant tumor patients in China [41], 1.32 times GDP for non-small cell lung cancer patients in Jiangsu Province [40], and 1.90 times GDP from the supplier perspective [42]. Additionally, our findings were lower than the value of statistical life-based estimates of 1.5 times the GDP for the general Chinese population [43] and 1.22 times the GDP for Liaoning Province [44], as well as 2.76 times the GDP for extending lifespan in Liaoning [44]. They also fell below the £50,000 threshold set by NICE for end-stage interventions in the UK [45], and the WTP/QALY values for end-stage cancer patients in the US (USD 12,500 to 32,200) and Switzerland (96,150 Swiss Francs) [39]. The unique socioeconomic characteristics observed among patients in Xinjiang may be a key reason for their significantly lower WTP/QALY than in other regions. WTP/QALY valuations exhibited pronounced heterogeneity relative to Xinjiang’s 2024 per capita disposable income (CNY 30,899; USD 4,325.86) as reported by the Regional Bureau of Statistics [46]. Additionally, research data indicate [47] that females generally exhibit lower WTP/QALY levels than males, a disparity that may be more pronounced among cervical cancer patients due to the disease’s distinct gender specificity. Further investigation with larger sample sizes and more rigorous study designs is needed to validate the mechanisms and extent of gender-related influences on WTP/QALY.

This study found significant variations in WTP/QALY values across different health improvement scenarios and QALY changes [48], differing from other studies [49]. Patients preferred paying for quality of life improvement over lifespan extension, consistent with Zhang M et al. [50]. The observed discrepancy suggests that respondents’ status (patients vs. healthy individuals) may influence the evaluation outcomes: patient groups exhibited higher willingness to pay for quality of life improvement, while healthy populations placed greater emphasis on life-year extension even in hypothetical scenarios [51]. The potential causes of this phenomenon may include: Extending life without improving the disease state imposes a heavy economic burden on the family and significant stress on caregivers, leading patients to perceive lower benefits from such life extension [52]. Patients exhibit lower overall psychological dimension scores and poorer self-perceived health status, which heightens their desire for a fully healthy life [53].

Methodological variations in utility assessment instruments significantly influence WTP/QALY estimations [54]. Our comparative analysis of EQ-5D-5 L and EQ-VAS revealed divergent performance characteristics across intervention types: the EQ-5D-5 L demonstrated greater sensitivity in detecting multidimensional health improvements, producing significantly higher WTP/QALY estimates than EQ-VAS in such scenarios. We propose a decision framework [29] aligning instrument selection with intervention complexity and study phase to optimize cost-effectiveness analyses. EQ-5D-5 L is more sensitive in assessing improvements in quality of life and better reflects the impact of time horizons on WTP/QALY. Despite differences in WTP/QALY estimates between the two methods, EQ-5D-5 L is more suitable for WTP/QALY calculations given economic considerations and the current research landscape [51]. This study primarily used EQ-5D-5 L, with EQ-VAS as a supplementary analysis. The absence of a consistency test limits the reliability of directly comparing results from the two instruments; future studies may consider incorporating consistency tests to validate their comparability.

This study using a DCE found that cervical cancer patients prioritize quality of life improvement over lifespan extension when choosing between targeted and immunotherapy drugs. This preference aligns with findings from Switzerland [39], China [55], and Malaysia, as well as with Zhe Feng et al. [56, 57], but contrasts with Yue Yin et al.‘s research on NSCLC patients who prioritized survival [58]. Cervical cancer patients also preferred treatments with fewer side effects, similar to results from Brazil [59]. Median survival was used as the survival attribute, which is more intuitive than progression-free survival [60]. Incorporating insurance reimbursement rates simulated real-world decision-making, revealing that patients are willing to pay more out-of-pocket for significant quality of life improvements or fewer side effects, even with lower reimbursement rates [61]. Patients demonstrated a tiered decision-making pattern in treatment selection: while remaining price-sensitive towards basic therapies, they showed a higher preference for innovative treatments that significantly improved quality of life or safety, even when facing increased out-of-pocket costs. These findings suggest that health insurance policies should consider providing higher reimbursement rates for innovative therapies with demonstrated clinical advantages, thereby optimizing the allocation of healthcare funds.

Patients exhibit a preference for improvements in quality of life over mere extensions in life expectancy, both in single-attribute and multi-attribute contexts. This finding can be elucidated by Prospect Theory [62] and Loss Aversion Theory [63]. According to Prospect Theory, decision-making is contingent upon changes relative to a reference point, rather than absolute gains or losses. For cervical cancer patients, their current quality of life serves as a pivotal reference point. Should their current quality of life be sub-optimal, they are more inclined to opt for treatment plans that significantly enhance their quality of life, as opposed to those that merely prolong life expectancy. This aligns with Loss Aversion Theory: achieving full health is perceived as a substantial gain, whereas extending life expectancy while maintaining the current disease state may entail potential losses, such as the economic burden of treatment, side effects, or psychological stress. Consequently, patients are willing to pay more to avoid further deterioration in their quality of life, as evidenced by their higher WTP for quality of life improvements compared to life expectancy extensions. This underscores the necessity of incorporating both quality of life and life expectancy into decision-making frameworks for health insurance coverage and resource allocation, rather than relying solely on life expectancy as the basis for decision-making [54].

Importantly, we found that most patients were unaware of how to participate in drug selection actively, often relying on doctors’ recommendations rather than their preferences. This reflects the “doctor-led” decision-making model prevalent in the Chinese healthcare system [64], where patients typically depend on doctors’ advice and are less involved in treatment decisions. Patients may exhibit a more passive decision-making style during treatment, due to illness, psychological factors, and other reasons, and are more likely to rely on physicians when selecting treatment options. In comparison to patients in Western countries, cervical cancer patients in China show less awareness and autonomy in drug choices, underscoring the need for improved doctor-patient communication and enhanced patient involvement in clinical decisions. In reality, regardless of whether cancer patients wish to participate in treatment decision-making, clinicians must select the most appropriate treatment options based on the specific circumstances of each patient [52].

This study offers critical insights into cervical cancer patients’ treatment preferences and health valuation in Xinjiang, though key limitations must be acknowledged. First, while interviewer-administered surveys with rigorous training protocols addressed local health literacy challenges, residual social desirability bias may persist in preference reporting [6]. Second, CVM and DCE design carry inherent hypothetical bias risks that could inflate WTP/QALY estimates, despite mitigation through cognitive pretesting and payment ladder calibration. Third, extreme WTP values present persistent interpretation challenges in distinguishing genuine preference intensity from measurement artifacts. Fourth, our sample size (*n* = 106) proved insufficient for advanced mixed logit modelling of preference heterogeneity [14]. This necessitated reliance on the conditional logit model to ensure robust population-level parameter estimates. This approach is methodologically defensible for small-sample discrete choice studies, prioritizing core preference stability over heterogeneity exploration. Fifth, educational disparities likely exacerbated cognitive burdens during complex health trade-off tasks, despite questionnaire simplification through iterative cognitive interviewing. These constraints collectively underscore the necessity for future research incorporating larger samples, enhanced validity safeguards in preference elicitation, and formal behavioral validation frameworks. We concur with methodological consensus [44] that advances in experimental design and sampling rigor remain essential for strengthening the policy relevance of patient-reported outcome studies in low-resource settings.

Nevertheless, this study represents the first systematic elucidation of treatment preferences and WTP among cervical cancer patients in Xinjiang. These findings provide crucial localized evidence for dynamic adjustments to oncology drug reimbursement lists and the development of differentiated payment policies in China’s western border regions. More importantly, our results offer key implications for clinical practice and health policy evaluation: substantial attention must be given to patients’ subjective treatment experiences and individualized value judgments beyond standardized health assessment tools. China’s healthcare decision-making paradigm is undergoing a significant shift from a “cost-dominance” approach to a “value-based” healthcare model, as demonstrated by the Center for Drug Evaluation of China’s National Medical Products Administration officially released the ‘Guidelines for the Application of Patient-Reported Outcomes in Drug Clinical Development (Trial)’, which established PROs as one of the key indicators in China’s drug evaluation and approval process [65]. Our research indicates that patients value improvements in their quality of life, suggesting that clinical decision-making processes should comprehensively integrate patients’ quality of life considerations and their WTP. This integration is essential to realize a patient-centered, value-based care system authentically. Future studies should explore how Xinjiang’s multicultural context and healthcare system characteristics influence patient autonomy, physician trust, and perceptions of medical technology, to better understand medical decision-making among cancer patients in culturally diverse regions.

## Conclusions

This study found that cervical cancer patients have lower WTP/QALY values for quality of life improvement and survival extension compared to other cancer patients in China. Factors such as quality of life improvement scores, median survival, incidence of severe adverse reactions, drug prices, and insurance reimbursement rates significantly impact patients’ treatment choices and willingness to pay. However, patients’ awareness of participating in treatment decisions is relatively low, with a heavy reliance on doctors’ recommendations. Therefore, the current healthcare system should place greater emphasis on patient preferences, enhance doctor-patient communication, and encourage more active patient involvement in treatment decisions to improve treatment outcomes and overall satisfaction.

## Supplementary Information

Below is the link to the electronic supplementary material.

Supplementary Material 1

## Data Availability

All data generated or analyzed during this study are included in this published article [and its supplementary information files].
