# Supplementary material for: Patient willingness to pay and preference for cervical cancer treatments among middle- and low-income populations in Xinjiang
Source: J Patient Rep Outcomes. 2025 Aug 21;9:107. doi: 10.1186/s41687-025-00938-6 (PMC12370612; doi:10.1186/s41687-025-00938-6)

**Supplementary File**

**Patient Preferences and Willingness to Pay for Cervical Cancer Treatments Among Middle- and Low-Income Populations in Xinjiang**

**Content**

[Appendix 1 Flowchart of Contingent Valuation Method Scenario Development 1](#_Toc176779404)

[Appendix 2 Determination of Attributes and Levels for Discrete Choice Experiment 2](#_Toc176779405)

[Appendix 3 Questionnaire for Willingness to pay and medication preference of cervical cancer patients 6](#_Toc176779406)

[Appendix 4 Box Plot of WTP per QALY and Frequency Distribution of WTP Values 17](#_Toc176779407)

# Appendix 1 Flowchart of Contingent Valuation Method Scenario Development

**Assess** current health status

(EQ-5D; EQ-VAS)

**Scenario 1:**

Perfect health

**Scenario 2:**

Current health

**Drug 1:**

Maintain for 5 years

**Drug 3:**

Gain 5 unadjusted life-years

**Drug 2:**

Maintain for 10 years years

**Drug 4:**

Gain 5 unadjusted life-years years

**Assess** willingness to pay (WTP)

(Payment card)

**Calculate** WTP and WTP/QALY for both scenarios

# Appendix 2 Determination of Attributes and Levels for Discrete Choice Experiment

Based on a systematic review of discrete choice experiments in oncology treatment, key attributes for targeted and immunotherapy drugs were identified, encompassing safety, efficacy, and cost dimensions. Regarding efficacy evaluation, both life extension and quality of life were considered. The quality of life attribute was derived from a study conducted in Henan province, which documented Quality Adjusted Life Years (QALY) losses ranging from 0.05 to 0.26 among cervical cancer patients (1). To more accurately capture patient preferences for QoL improvement, the study operationalized quality of life improvement as incremental increases of 5, 15, and 25 points on the Visual Analog Scale (VAS). This methodological approach was designed to account for patients' interpretability of scoring systems while systematically evaluating preferences across varying magnitudes of QoL improvement, thereby enhancing the scientific validity and comprehensive nature of the investigation.

**Table A2-1 Key Attributes in Cancer Discrete Choice Experiments: Study Summary**

| Systematic Review of Attributes in Cancer Discrete Choice Experiments | **Reference** | **Attributes** |
| --- | --- | --- |
|  | S. Jiang(2) | Overall survival |
|  |  | Progression-free survival |
|  |  | Adverse effects |
|  |  | Cost |
|  | D. R. Bien(3) | Effectiveness |
|  |  | Adverse effects |
|  |  | Quality of life |
|  |  | Cost |
|  | Hannah Collacott (4) | Overall survival |
|  |  | Progression-free survival |
|  |  | Quality of life |
|  |  | Cost |

**Determination of Safety and Efficacy Attributes and Levels**

The determination of safety and efficacy levels was based on a comprehensive review of clinical trial literature for targeted and immunotherapy drugs. The safety attribute was operationalized as the incidence of Grade 3 or higher adverse reactions, as established through this review process. For efficacy assessment, three key parameters were selected: median survival time, progression-free survival, and response rate, all of which were identified as clinically meaningful endpoints in the examined trials.

**Table A2-2 Review of Clinical Trial Outcomes for Targeted and Immunotherapies**

| **Attribute** | **Range** | **Reference** |
| --- | --- | --- |
| Median OS | 3.7-17 months | Chakor Vora(5),  Luopei Guo(6),  Sherer M V(7),  F.J. Crowley(8),  D. E. Watkins(9) |
| Grade 3 or 4 toxicities | 3%-23.5% |  |
| Median PFS | 3.52-10.9 months |  |
| Response rate | 5%-61% |  |

**Determination of Cost Attributes and Levels**

The price attribute was determined through a comprehensive review of cervical cancer-targeted and immunotherapy drugs available in China, with reference to the National Medical Products Administration's drug review center (https://www.cde.org.cn/) and various pharmaceutical company websites, as detailed in Table 3. To optimize patient understanding of drug pricing, the cost presentation was structured as monthly expenses rather than total treatment course costs, accounting for substantial variability in treatment duration and potential cost fluctuations among individuals. This monthly cost framework was specifically designed to facilitate more accurate financial burden assessment and support informed decision-making regarding treatment budgets.

Three distinct price levels (3,000 CNY/month, 8,000 CNY/month, and 12,000 CNY/month) were established based on actual market pricing data, combined with clinical input from oncology experts and patient feedback. These levels were carefully selected to reflect the full spectrum of available treatment options, from relatively affordable therapies to higher-cost premium regimens, ensuring representation of diverse economic considerations in the study design.

**Table A2-3 Approved Targeted Therapies and Immunotherapies for Cervical Cancer in China**

| **No.** | **Drug Name** | **Brand Name** | **Manufacturer** | **Approval Status** | **Price** | **Drug Type** |
| --- | --- | --- | --- | --- | --- | --- |
| 1 | Bevacizumab | Avastin® | Roche | FDA,  NMPA | 1500 CNY/vial  (4ml:0.1g) | Targeted Drug |
| 2 | Sepalizumab | Yutuo® | Hengrui Bio | NMPA | 3300 CNY/vial  (120mg:4ml) | Immunotherapy |
| 3 | Camrelizumab | Airuika® | Hengrui Pharma | NMPA | 2576.44 CNY/vial  (200mg/vial) | Immunotherapy |
| 4 | Cadonilimab | Kaitanni® | Akeso | NMPA | 6166 CNY/vial  (125mg:10ml) | Immunotherapy |

**Expert Interviews**

Thirteen oncology experts were interviewed to evaluate nine preliminary drug attributes and their respective levels. The expert panel comprised 12 clinicians and 1 clinical pharmacist, including 8 experts with over 10 years of experience and 4 experts with 5-10 years of experience. During the interviews, experts were asked to: (1) identify the five most influential attributes affecting cervical cancer patients' drug choices, (2) discuss any additional relevant attributes not initially considered, and (3) evaluate the appropriateness of the proposed attribute levels.

Through this expert consultation process, five key attributes were identified based on comprehensive rankings: drug cost, medical reimbursement rate, incidence of severe adverse reactions, median survival time, and quality of life improvement. Expert feedback indicated that "route of administration" and "treatment method" were unsuitable as primary study attributes, resulting in their exclusion from the final selection. Furthermore, based on expert recommendations, the reimbursement rate levels were adjusted from the initial 30%, 50%, and 70% to more clinically relevant levels of 50%, 70%, and 90%.

**Patient Focus Group Discussions**

Focus group discussions were conducted with 15 cervical cancer patients (mean age: 55 years), representing diverse disease stages (I-IV) and treatment experiences encompassing both conventional therapies (surgery, radiotherapy, chemotherapy) and novel approaches (targeted therapy, immunotherapy).

Analysis of these discussions revealed five priority attributes for patients: quality of life improvement, incidence of severe adverse reactions, median survival time, drug cost, and medical reimbursement rate. Particular patient emphasis was observed regarding efficacy-related attributes such as progression-free survival, with participants providing substantive insights into how these factors influenced their treatment decision-making processes.

To ensure clinical validity and experimental robustness, attribute levels were carefully calibrated through an iterative process incorporating both expert clinical input and patient perspectives. This refinement process addressed several key considerations: (1) inadequate representation of clinical reality in preliminary survival time parameters, and (2) the need to encompass both typical and extreme clinical values. Consequently, final attribute levels were established as follows: median survival time (10, 15, and 22 months) and incidence of severe adverse reactions (Grade 3+, 10%, 30%, and 50%). The complete set of finalized attributes and levels is presented in Table 4.

**Table 4 Discrete Choice Experiment Attribute Levels and Definitions**

| **Attribute** | **Level** | **Definition** |
| --- | --- | --- |
| Median Survival Time | 10 months; 15 months; 22 months | The survival time of half of the patients after receiving treatment |
| Quality of Life Improvement | 5 points; 15 points; 25 points | The impact of treatment on patients' quality of life (assessed by VAS) |
| Incidence of Severe Adverse Reactions | 10%; 30%; 50% | The probability of patients experiencing severe adverse reactions (grade 3 and above) |
| Insurance Reimbursement Ratio | 50%; 70%; 90% | The reimbursement ratio if the drug is covered by health insurance |
| Drug Cost | 3000 CNY/month; 8000 CNY/month; 12000 CNY/month | The pricing of the drug after its market release, representing the monthly treatment cost |

**Reference:**

1. Wu Q, Jia M, Chen H, Zhang S, Liu Y, Prem K, et al. The economic burden of cervical cancer from diagnosis to one year after final discharge in Henan Province, China: A retrospective case series study. PLoS One. 2020;15(5):e0232129.

2. Jiang S, Ren R, Gu Y, Jeet V, Liu P, Li S. Patient Preferences in Targeted Pharmacotherapy for Cancers: A Systematic Review of Discrete Choice Experiments. Pharmacoeconomics. 2023;41(1):43-57.

3. Bien DR, Danner M, Vennedey V, Civello D, Evers SM, Hiligsmann M. Patients' Preferences for Outcome, Process and Cost Attributes in Cancer Treatment: A Systematic Review of Discrete Choice Experiments. Patient. 2017;10(5):553-65.

4. Collacott H, Soekhai V, Thomas C, Brooks A, Brookes E, Lo R, et al. A Systematic Review of Discrete Choice Experiments in Oncology Treatments. The Patient - Patient-Centered Outcomes Research. 2021;14(6):775-90.

5. Vora C, Gupta S. Targeted therapy in cervical cancer. ESMO Open. 2018;3(Suppl 1):e000462.

6. Guo L, Hua K. Cervical Cancer: Emerging Immune Landscape and Treatment. Onco Targets Ther. 2020;13:8037-47.

7. Sherer MV, Kotha NV, Williamson C, Mayadev J. Advances in immunotherapy for cervical cancer: recent developments and future directions. Int J Gynecol Cancer. 2022;32(3):281-7.

8. Crowley FJ, O'Cearbhaill RE, Collins DC. Exploiting somatic alterations as therapeutic targets in advanced and metastatic cervical cancer. Cancer Treat Rev. 2021;98:102225.

9. Watkins DE, Craig DJ, Vellani SD, Hegazi A, Fredrickson KJ, Walter A, et al. Advances in Targeted Therapy for the Treatment of Cervical Cancer. J Clin Med. 2023;12(18).

# Appendix 3 Questionnaire for Willingness to pay and medication preference of cervical cancer patients

*This is a translated version of the questionnaire; the original is Chinese.*

Dear Cervical Cancer Patient:

Greetings! We are a research team from the Department of Pharmacy of the First Affiliated Hospital of Xinjiang Medical University. We are currently working on the willingness to pay for treatment and dosing preferences for targeted/immunologic drugs in cervical cancer patients.

The incidence of cervical cancer in Xinjiang is at a high level in the country, causing great suffering to many patients. We are well aware of the hardships and challenges involved in the treatment of cervical cancer, and we hope to use this study to find out how much you are willing to pay for the treatment of cervical cancer and your preferences for different targeted/immunologic drugs.

Your participation will not only help healthcare providers and pharmaceutical companies better understand patients' needs but also drive the optimization and improvement of future cervical cancer treatment options. Your voice is critical to medical decision-making! By participating in this study, you are not only fighting for better treatment options for yourself but also contributing to the future of all cervical cancer patients!

The questionnaire will take about 20-30 minutes to complete, and you can contact our surveyors if you have any questions!

Thank you for your support and participation. We wish you the best of luck in your treatment and hope to hear your voice!

Research Team,

1.Please confirm with the investigator and fill in the investigator's number：

2.Which hospital are you currently attending?

○ The First Affiliated Hospital of Xinjiang Medical University
○ The Affiliated Cancer Hospital of Xinjiang Medical University
○ Other _________________

3.Please fill in your hospitalization number (please confirm and verify the accuracy of the hospitalization number with the investigator)

4.What is your approximate average annual income?

○ Less than 12,000 RMB
○ 12,000 RMB - 30,000 RMB
○ 30,000 RMB - 50,000 RMB
○ 50,000 RMB - 70,000 RMB
○ 70,000 RMB - 100,000 RMB
○ More than 100,000 RMB
○ Unable to work due to illness, no income
○ No job, no income

5.To what extent does your family or friends support you in your treatment for cervical cancer?

○ Fully support
○ Mostly support
○ Partially support
○ Hardly support
○ Do not support at all

**Under each heading, please tick the ONE box that best describes your health today.**

6.MOBILITY

 I have no problems in walking about

 I have slight problems in walking about

 I have moderate problems in walking about

 I have severe problems in walking about

 I am unable to walk about

7.SELF-CARE

 I have no problems washing or dressing myself

 I have slight problems washing or dressing myself

 I have moderate problems washing or dressing myself

 I have severe problems washing or dressing myself

 I am unable to wash or dress myself

8.USUAL ACTIVITIES (e.g. work, study, housework, family or leisure activities)

 I have no problems doing my usual activities

 I have slight problems doing my usual activities

 I have moderate problems doing my usual activities

 I have severe problems doing my usual activities

 I am unable to do my usual activities

9.PAIN / DISCOMFORT

 I have no pain or discomfort

 I have slight pain or discomfort

 I have moderate pain or discomfort

 I have severe pain or discomfort

 I have extreme pain or discomfort

10.ANXIETY / DEPRESSION

 I am not anxious or depressed

 I am slightly anxious or depressed

 I am moderately anxious or depressed

 I am severely anxious or depressed

 I am extremely anxious or depressed

11.We would like to know how good or bad your health is TODAY. This scale is numbered from 0 to 100. 100 means the best health you can imagine. 0 means death. Please mark an X on the scale to indicate how your health is TODAY. Now, write the number you marked on the scale in the box below.


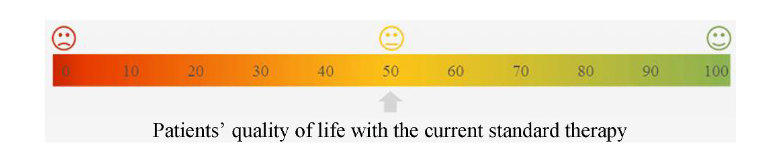


1. Imagine a "new treatment" that can completely eliminate your cervical cancer symptoms, returning you to full health (no difficulty moving, washing, dressing, doing daily activities, no pain, discomfort, anxiety, or depression/essentially a perfect score of 100). If this treatment could keep you in perfect health for 5 years, how much would you be willing to pay for it? (One-time payment)

○ More than 893,580 yuan

○ 446,790-893,580 yuan

○ 402,111-446,790 yuan

○ 357,432-402,111 yuan

○ 312,753-357,432 yuan

○ 268,074-312,753 yuan

○ 223,395-268,074 yuan

○ 178,716-223,395 yuan

○ 134,037-178,716 yuan

○ 89,358-134,037 yuan

○ 44,679-89,358 yuan

○ 17,872-44,679 yuan

○ 8,936-17,872 yuan

○ Less than 8,936 yuan

○ I am not willing to pay _________________

1. Imagine a "new treatment" that can completely eliminate your cervical cancer symptoms, returning you to full health (no difficulty moving, washing, dressing, doing daily activities, no pain, discomfort, anxiety, or depression/essentially a perfect score of 100). If this treatment could keep you in perfect health for 10 years, how much would you be willing to pay for it? (One-time payment)

○ More than 893,580 yuan

○ 446,790-893,580 yuan

○ 402,111-446,790 yuan

○ 357,432-402,111 yuan

○ 312,753-357,432 yuan

○ 268,074-312,753 yuan

○ 223,395-268,074 yuan

○ 178,716-223,395 yuan

○ 134,037-178,716 yuan

○ 89,358-134,037 yuan

○ 44,679-89,358 yuan

○ 17,872-44,679 yuan

○ 8,936-17,872 yuan

○ Less than 8,936 yuan

○ I am not willing to pay _________________

1. Imagine a "new treatment" that could extend your life by 5 years without improving your current health condition (as described in questions 6 to 10 or according to the score you gave in question 11). In this case, how much would you be willing to pay for this treatment? (One-time payment)

○ More than 893,580 yuan

○ 446,790-893,580 yuan

○ 402,111-446,790 yuan

○ 357,432-402,111 yuan

○ 312,753-357,432 yuan

○ 268,074-312,753 yuan

○ 223,395-268,074 yuan

○ 178,716-223,395 yuan

○ 134,037-178,716 yuan

○ 89,358-134,037 yuan

○ 44,679-89,358 yuan

○ 17,872-44,679 yuan

○ 8,936-17,872 yuan

○ Less than 8,936 yuan

○ I am not willing to pay _________________

1. Imagine a "new treatment" that could extend your life by 10 years without improving your current health condition (as described in questions 6 to 10 or according to the score you gave in question 11). In this case, how much would you be willing to pay for this treatment? (One-time payment)

○ More than 893,580 yuan

○ 446,790-893,580 yuan

○ 402,111-446,790 yuan

○ 357,432-402,111 yuan

○ 312,753-357,432 yuan

○ 268,074-312,753 yuan

○ 223,395-268,074 yuan

○ 178,716-223,395 yuan

○ 134,037-178,716 yuan

○ 89,358-134,037 yuan

○ 44,679-89,358 yuan

○ 17,872-44,679 yuan

○ 8,936-17,872 yuan

○ Less than 8,936 yuan

○ I am not willing to pay _________________

1. Are you familiar with targeted or immunotherapy drugs for cervical cancer?？

○ Very well informed
○ Informed
○ Somewhat informed
○ Almost uninformed
○ Completely uninformed

In this section, we aim to understand your preferences when choosing targeted or immunotherapy drugs for cervical cancer. Each question will present five attributes (see the image below). You will be shown three different drug options, each with these five attributes.

1. After reading the content in the image above, do you understand the attributes and levels set?

○ Completely understand
○ Mostly understand
○ Understand somewhat
○ Almost do not understand
○ Do not understand at al

1. When choosing targeted or immunotherapy drugs for your disease, please rank the importance of the following five attributes. You need to click the boxes in order of importance. [Ranking question: Please fill in the numbers in the brackets according to the order of importance]

[ ] Median survival: The time when 50% of patients survive after treatment
[ ] Improvement in quality of life: The extent to which the drug improves quality of life
[ ] Health insurance reimbursement rate: The reimbursement rate after the drug is included in the insurance directory
[ ] Incidence of serious adverse reactions (grade 3 or above): Includes high blood pressure (grade 3 or above), proteinuria (grade 3), gastrointestinal perforation (grade 3 and above), etc.
[ ] Drug cost: The market price of the drug after its launch

1. If you needed to choose a type of targeted therapy/immunotherapy drug for treating your condition, which drug would you choose?


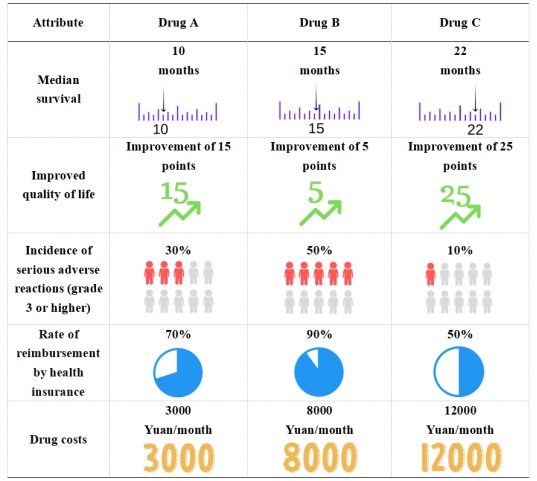


○ Drug A
○ Drug B
○ Drug C

1. If you needed to choose a type of targeted therapy/immunotherapy drug for treating your condition, which drug would you choose?


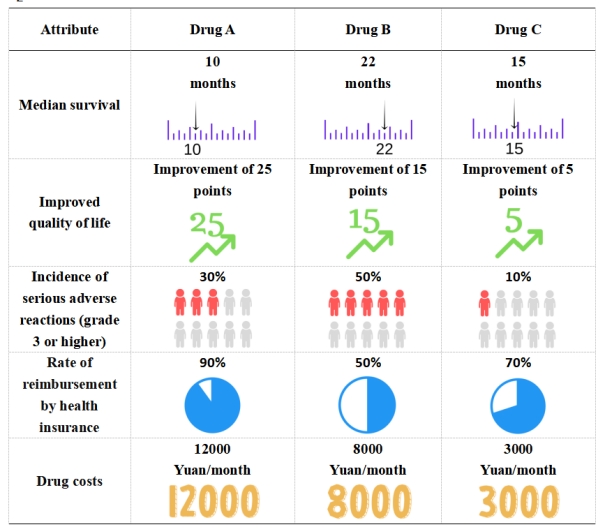


○ Drug A
○ Drug B
○ Drug C

1. If you needed to choose a type of targeted therapy/immunotherapy drug for treating your condition, which drug would you choose?


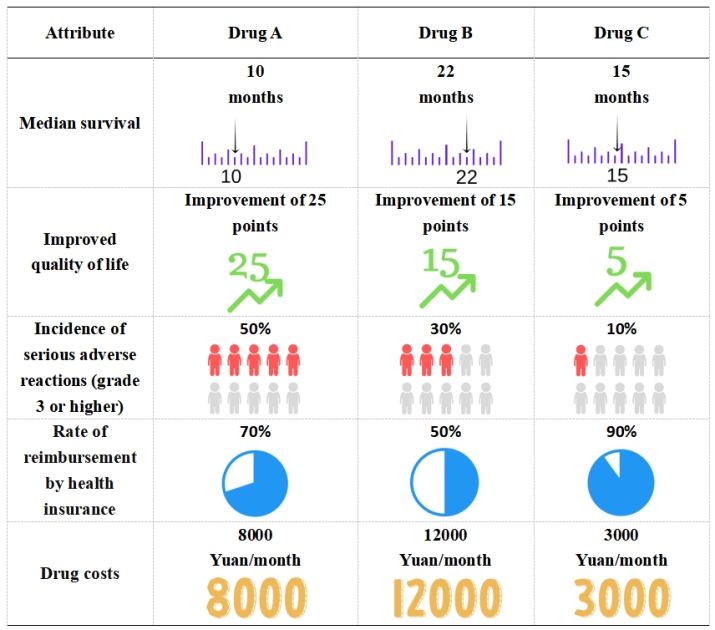


○ Drug A
○ Drug B
○ Drug C

1. If you needed to choose a type of targeted therapy/immunotherapy drug for treating your condition, which drug would you choose?


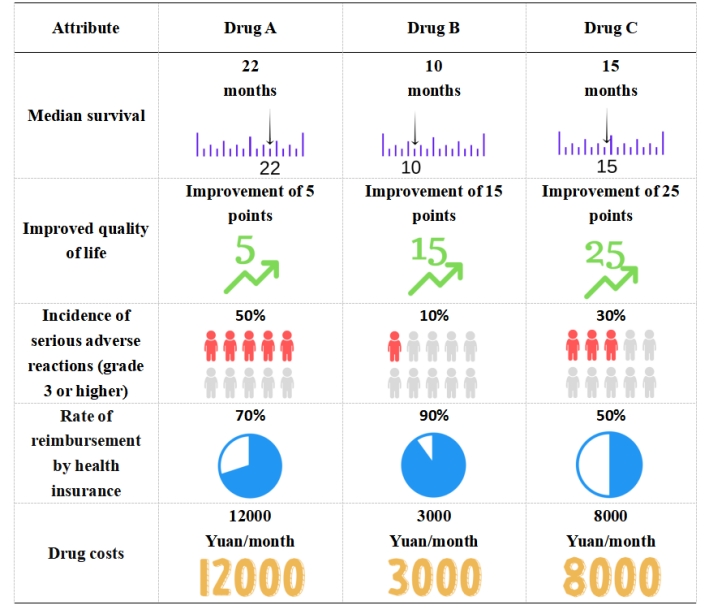


○ Drug A
○ Drug B
○ Drug C

1. If you needed to choose a type of targeted therapy/immunotherapy drug for treating your condition, which drug would you choose?


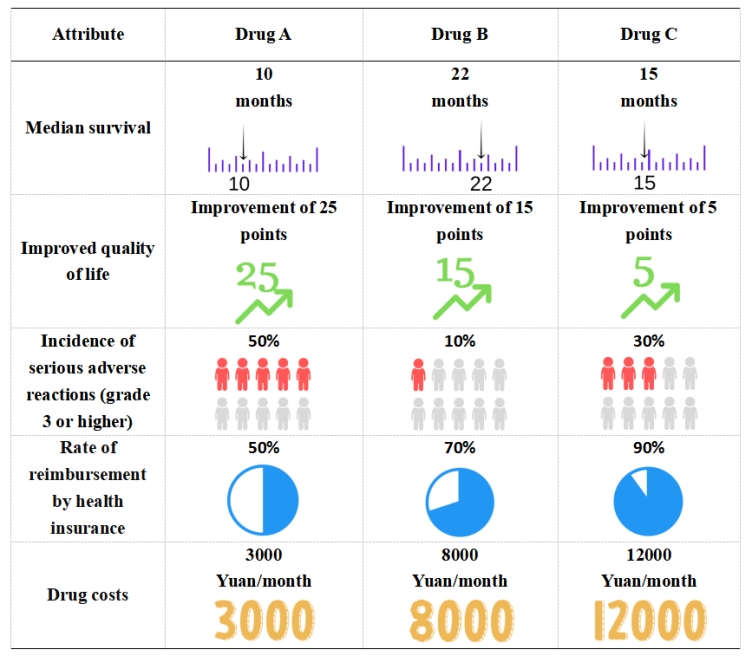


○ Drug A
○ Drug B
○ Drug C

1. If you needed to choose a type of targeted therapy/immunotherapy drug for treating your condition, which drug would you choose?


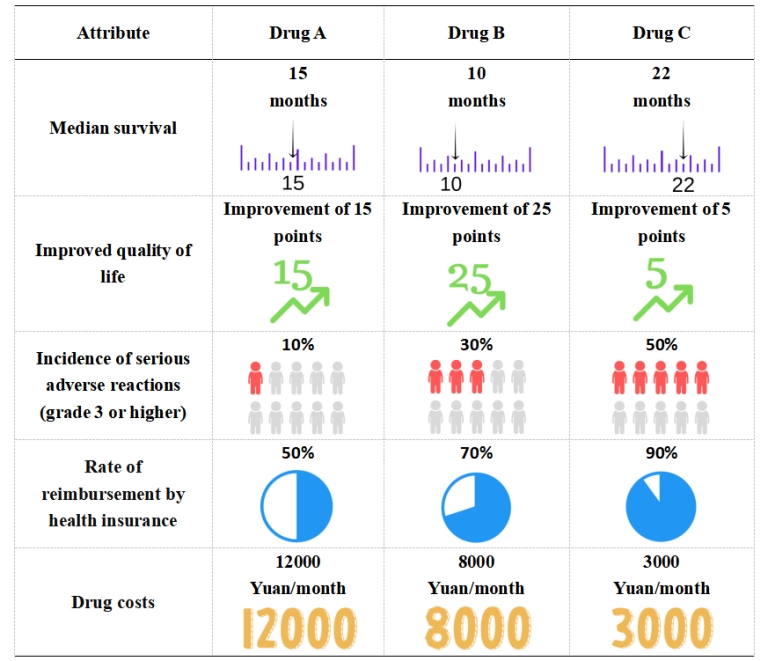


○ Drug A
○ Drug B
○ Drug C

1. If you needed to choose a type of targeted therapy/immunotherapy drug for treating your condition, which drug would you choose?


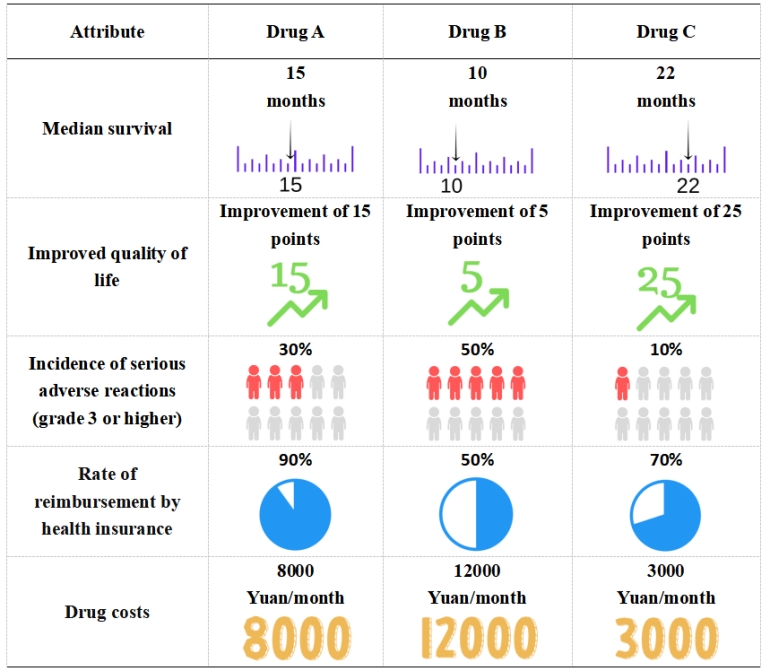


○ Drug A
○ Drug B
○ Drug C

1. If you needed to choose a type of targeted therapy/immunotherapy drug for treating your condition, which drug would you choose?


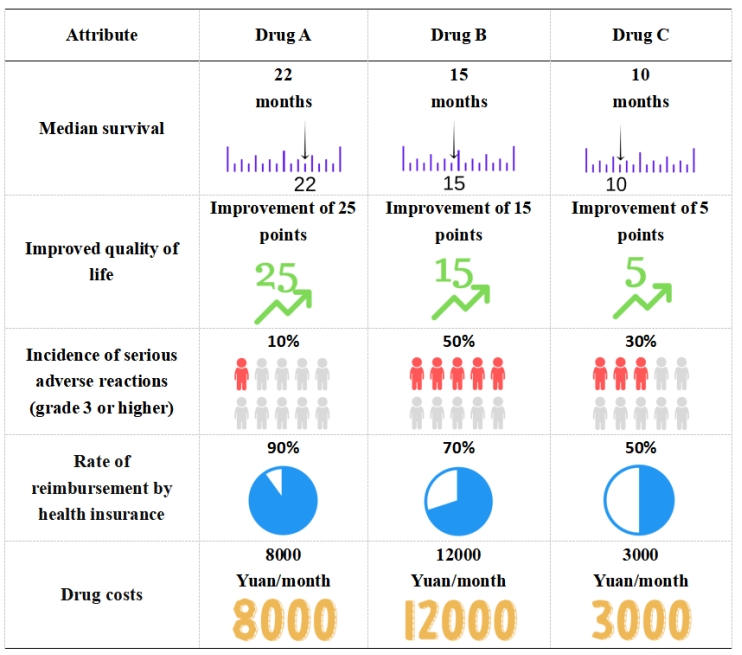


○ Drug A
○ Drug B
○ Drug C

1. If you needed to choose a type of targeted therapy/immunotherapy drug for treating your condition, which drug would you choose?


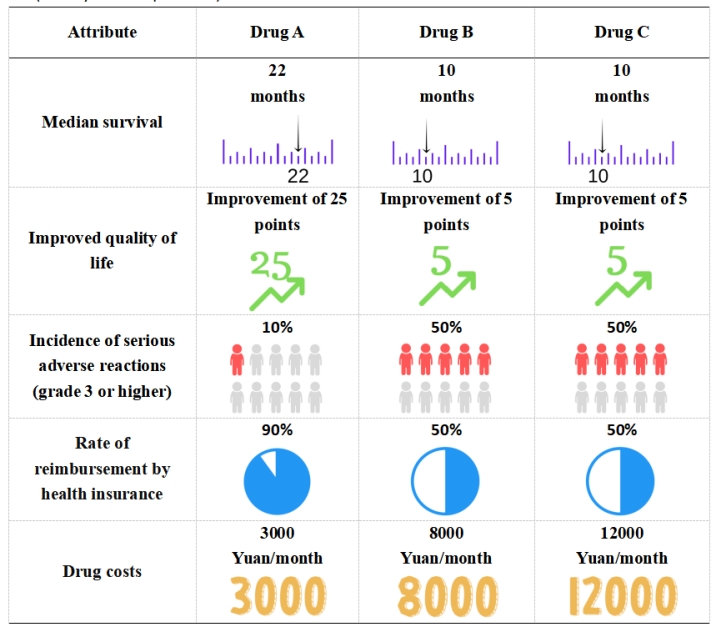


○ Drug A
○ Drug B
○ Drug C

1. If you needed to choose a type of targeted therapy/immunotherapy drug for treating your condition, which drug would you choose?


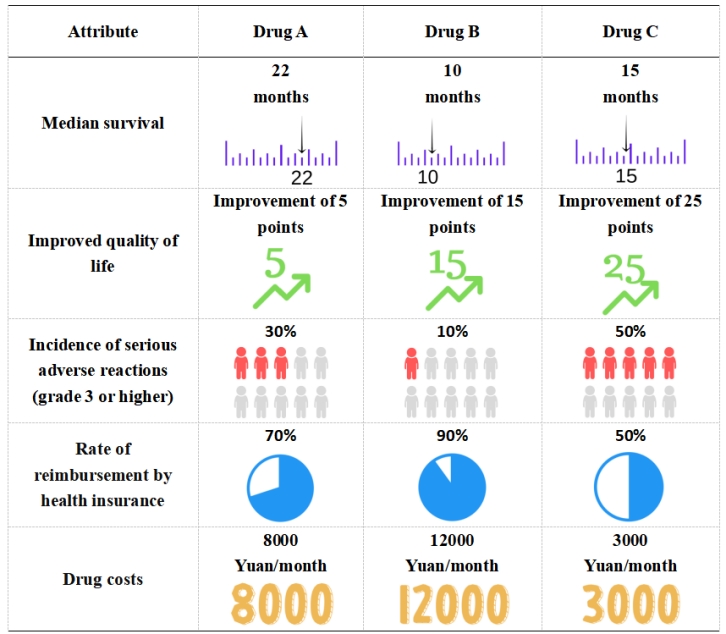


○ Drug A
○ Drug B
○ Drug C

1. If you needed to choose a type of targeted therapy/immunotherapy drug for treating your condition, which drug would you choose？

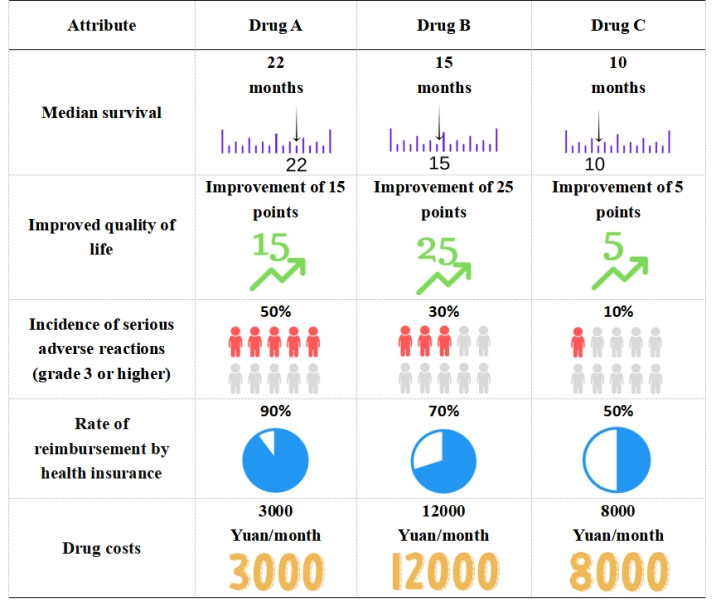


○ Drug A
○ Drug B
○ Drug C

# Appendix 4 Box Plot of WTP per QALY and Frequency Distribution of WTP Values

**Figure 1 WTP/QALY for Quality of Life Improvement**


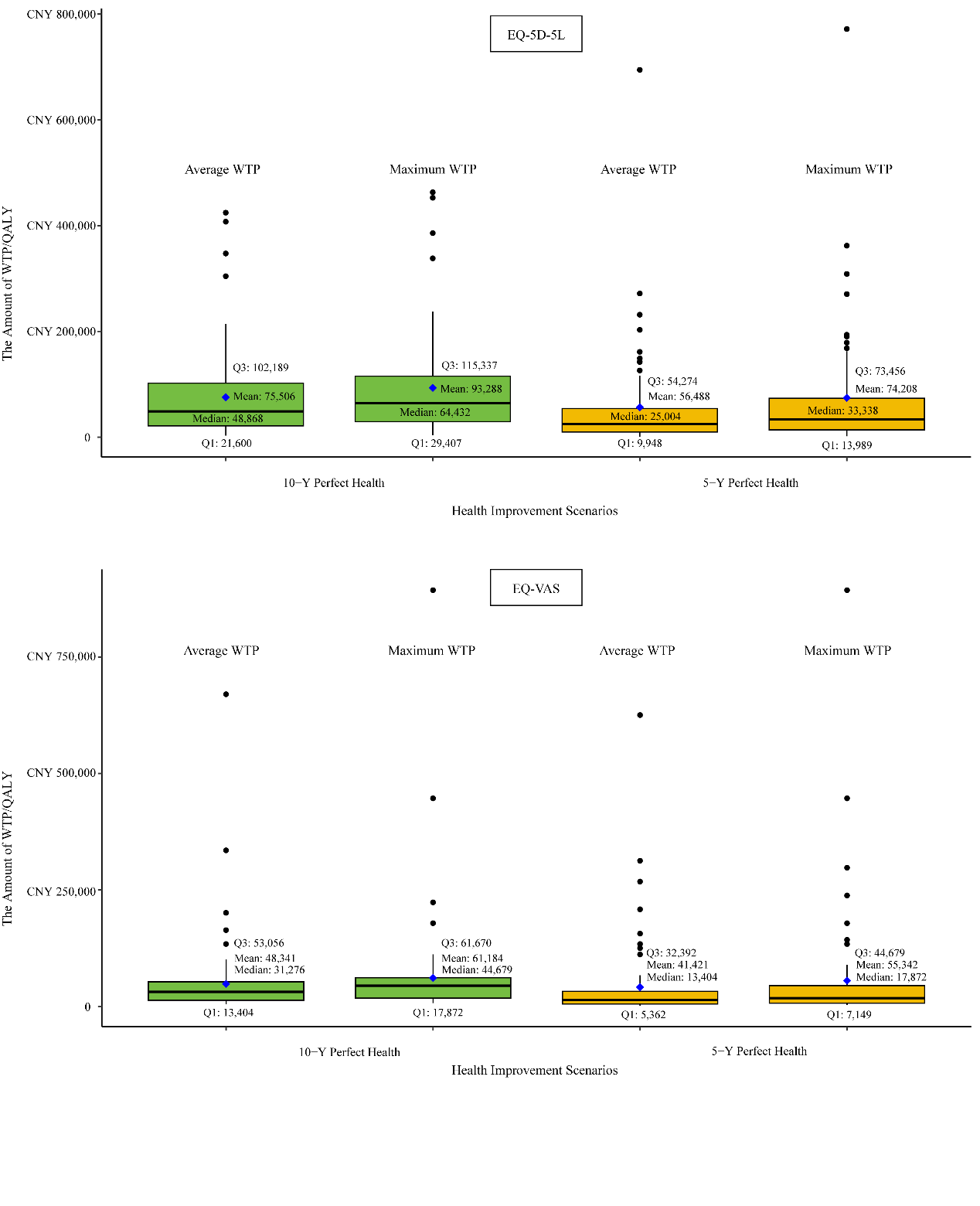


Note: Black dots represent outliers or extreme values.

"10-Y Perfect Health" refers to 10 years of perfect health, and "5-Y Perfect Health" refers to 5 years of perfect health.

**Figure 2 WTP/QALY for Unadjusted Life Years**


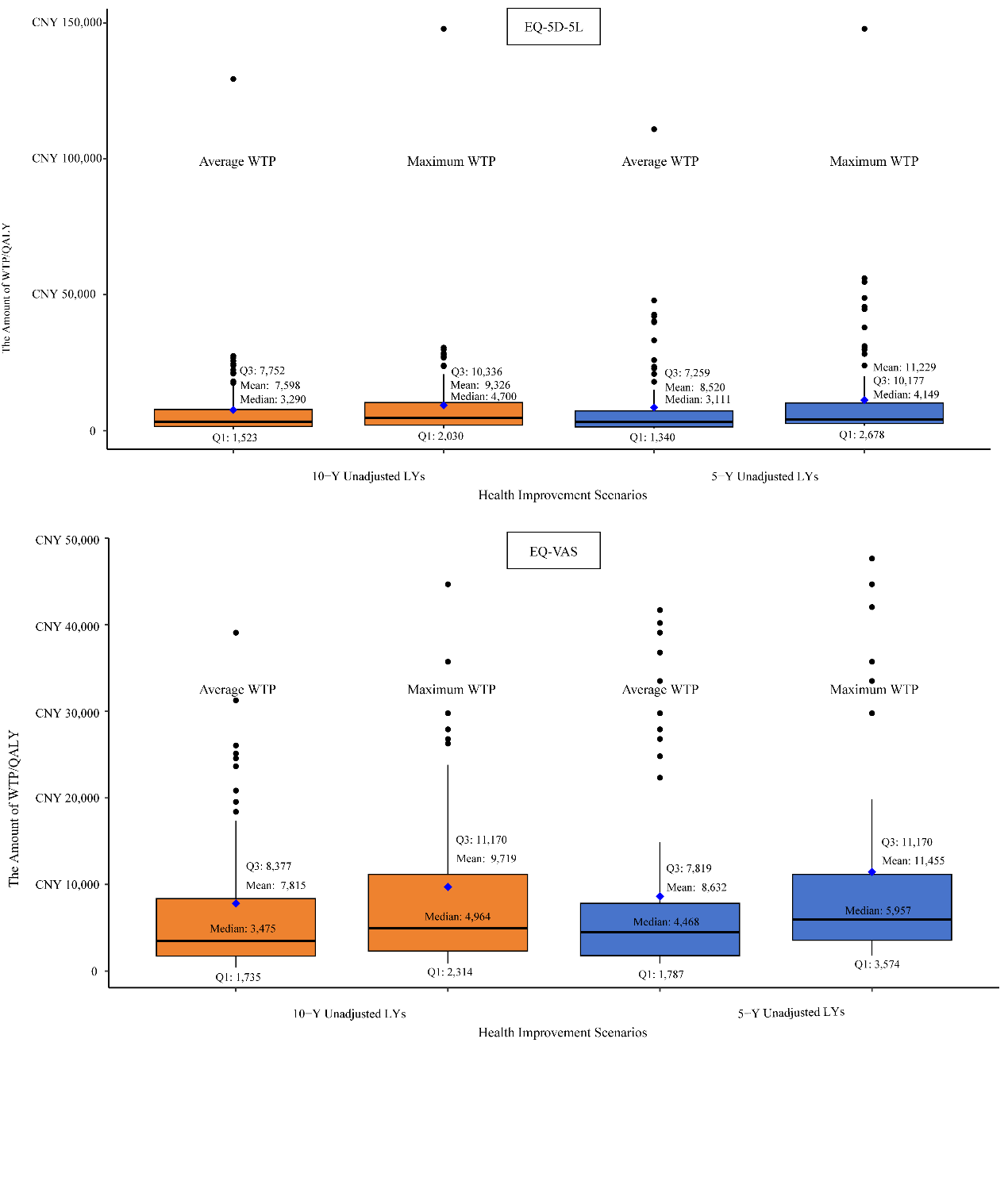


Note: Black dots represent outliers or extreme values.

"10-Y Unadjusted LYs " refers to a 10-year survival gain without change in health utility, and "5-Y Unadjusted LYs " refers to a 5-year survival gain without change in health utility.

**Figure 3 Frequency Distribution of Willingness to Pay (WTP)**


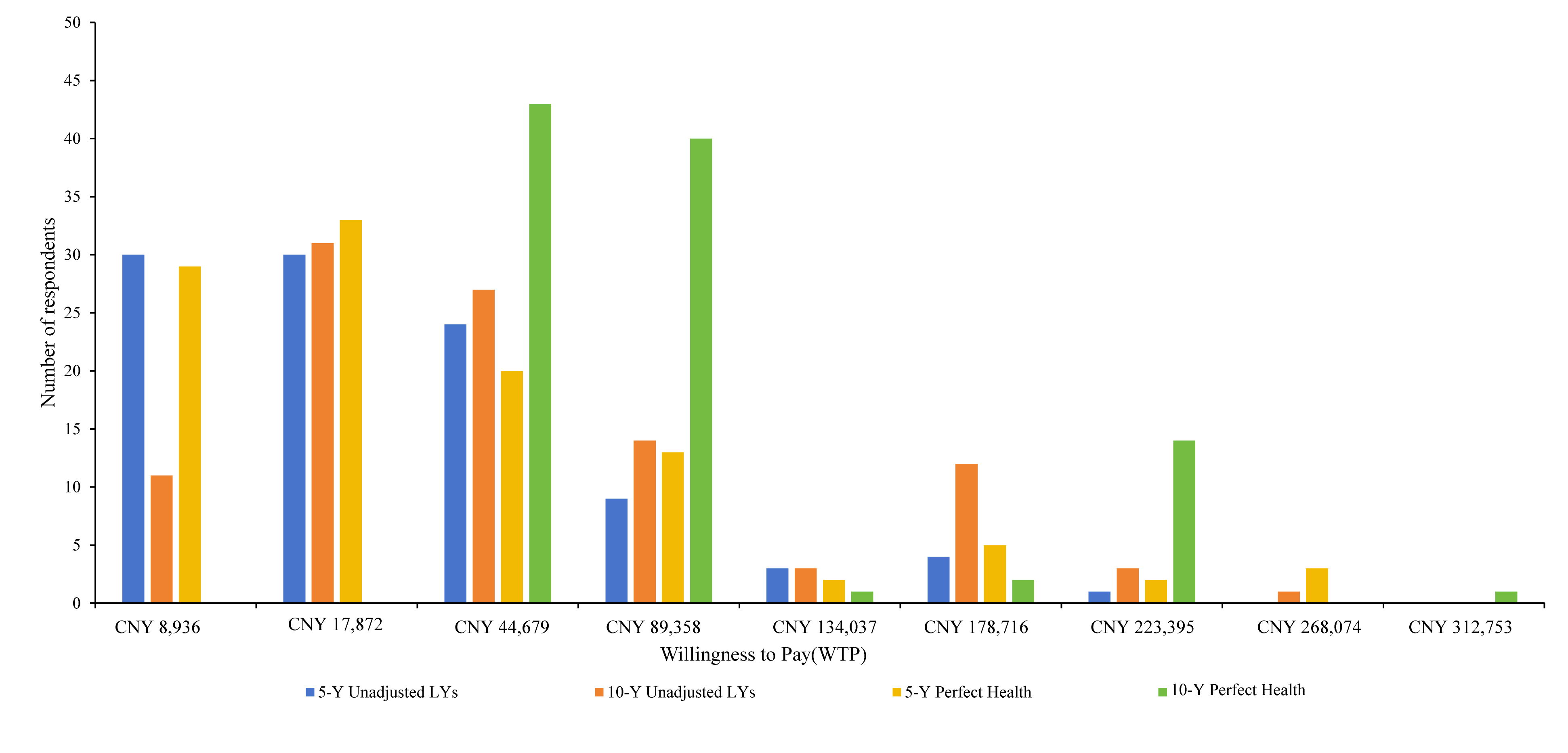

Supplement: Supplementary file 1 — Supplementary Material 1 [file 41687_2025_938_MOESM1_ESM.docx]
